# Erythrophagocytosis by Microglia/Macrophage in Intracerebral Hemorrhage: From Mechanisms to Translation

**DOI:** 10.3389/fncel.2022.818602

**Published:** 2022-02-14

**Authors:** Jiaxin Liu, Zhiyuan Zhu, Gilberto Ka-Kit Leung

**Affiliations:** ^1^Department of Surgery, LKS Faculty of Medicine, The University of Hong Kong, Queen Mary Hospital, Hong Kong, Hong Kong SAR, China; ^2^Department of Functional Neurosurgery, The National Key Clinical Specialty, The Engineering Technology Research Center of Education Ministry of China, Guangdong Provincial Key Laboratory on Brain Function Repair and Regeneration, The Neurosurgery Institute of Guangdong Province, Guangzhou, China; ^3^Zhujiang Hospital, Southern Medical University, Guangzhou, China

**Keywords:** erythrophagocytosis, efferocytosis, macrophage, microglia, intracerebral hemorrhage, hematoma, phagocytosis

## Abstract

Intracerebral hemorrhage (ICH) is a devastating condition characterized by hematoma related mass effect. Microglia/macrophage (M φ) are rapidly recruited in order to remove the red blood cells through erythrophagocytosis. Efficient erythrophagocytosis can detoxify hemolytic products and facilitate neurological recovery after ICH. The underlying mechanisms include modulation of inflammatory response and oxidative stress, among others. It is a dynamic process mediated by a cascade of signal transduction, including “find-me” signals, “eat-me” signals and a set of phagocytotic receptors-ligand pairs that may be exploited as therapeutic targets. This review summarizes mechanistic signaling pathways of erythrophagocytosis and highlights the potential of harnessing M φ-mediated phagocytosis for ICH treatment.

## Introduction

In intracerebral hemorrhage (ICH), the rupture of brain vessels results in the accumulation of millions of red blood cells (RBCs) within brain parenchyma. Surgical excavation of hematoma is not recommended for most ICH cases due to questionable clinical benefits and adverse effects of surgery (Hemphill et al., 2015). Hemolysis within the hematoma may cause significant secondary injuries and irreversible neurological deficits due to the toxic properties of hemolytic products (Ziai, 2013). Microglia and monocyte-derived macrophage (M φ) are rapidly recruited at the bleeding site and may aid hematoma resolution by phagocytosing RBCs through erythrophagocytosis (Zhao et al., 2009; Chang C. F. et al., 2018; Jing et al., 2019). This detoxication process helps alleviate the brain injuries results from secondary detrimental process such as neuroinflammation and oxidative stress. Increasing number of studies have investigated the mechanistic signaling and beneficial effects of erythrophagocytosis in ICH (Chang C. F. et al., 2018; Chang et al., 2020; Xu et al., 2020). However, this endogenous erythrophagocytosis tends to be incomplete, and hemolytic products-triggered brain damage remains common and detrimental to recovery after ICH. Thus, enhancing endogenous erythrophagocytosis is an important strategy for ICH treatment.

In this review, we highlight the therapeutic values of targeting erythrophagocytosis in ICH. Firstly, we introduce the neurotoxicity of hematoma and the role of M φ-mediated erythrophagocytosis in hematoma clearance and ICH recovery. Then, we describe the dynamic process of phagocytosis, focusing on the essential membrane receptors in M φ. We further depict how erythrophagocytosis may be modulated by microenvironmental factors, including inflammatory cytokines and oxidative stress. Finally, we summarize the critical nuclear factors regulating erythrophagocytosis that could serve as druggable targets. The aim is to inform future pre-clinical and clinical studies on accelerating hematoma resolution as a means of improving patient outcomes in ICH.

## Neurotoxicity of Hematoma

**FIGURE 1:**
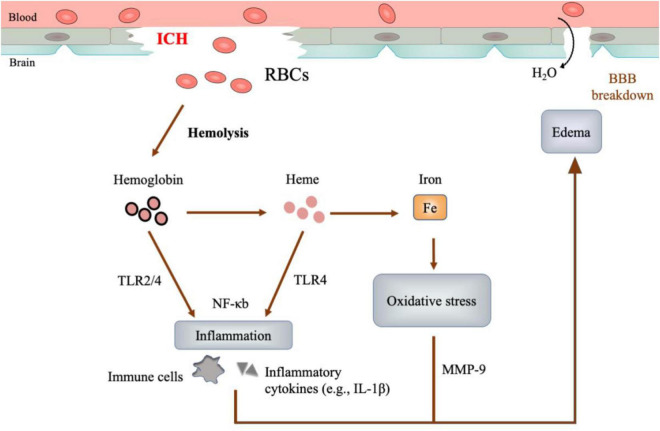
Neurotoxicity of hematoma. After ICH, red blood cells (RBCs) would undergo hemolysis and degrade into hemoglobin, heme and iron consequently. These hemolysis products mediate cascades of inflammation, oxidative stress, and perihematomal edema, causing detrimental injury to the brain.

The extravasated blood plays a critical role in ICH pathology. In acute ICH, the blood cells and other blood components from the ruptured vessels rapidly accumulate in the brain parenchymal, forming a hematoma with mass effect that can destroy the cerebral architecture (mass effect) (Xi et al., 2006). Subsequently, hemolysis occurs, releasing neurotoxic degradation products (hemoglobin, heme, and iron). These substances could cause detrimental effects on brain tissue (Figure 1; Wagner et al., 1996; Nakamura et al., 2005; Xue and Yong, 2020):

– **Inflammation** is represented by the rapid recruitment of immune cells and the inflammatory cytokines in perihematomal regions (Loftspring et al., 2009; Zia et al., 2012). The hemolysis products, especially hemoglobin and heme, may function as the ligands of Toll-like receptor 4 (TLR-4), which could activate proinflammatory M φ and elevate the levels of proinflammatory cytokines (Figueiredo et al., 2007; Kwon et al., 2015; Lan et al., 2017).
– **Oxidative stress** is featured by the accumulation of reactive oxygen species (ROS), which oxidize DNA, protein, and lipid, causing tissue damage (Marnett et al., 2003). Iron, generated from the heme degradation, can catalyze the well-studied Haber-Weiss reaction, yielding overwhelming ROS and resulting in oxidative stress (Wu et al., 2003; Nakamura et al., 2005).
– **Edema** develops as early as hours and peaks at a range of 10-20 days in patients after ICH (Xi et al., 2006). At the early phase (< 3 days), thrombin and serum proteins extruded from the hematoma are the leading cause of edema. At the later stage (> 3 days), the hemolysis products precipitate delayed edema (Wagner et al., 1996; Urday et al., 2015). Of note, the TLR-dependent inflammation by hemoglobin and heme, as well as the Matrix metallopeptidase 9 (MMP-9) activation and the oxidative stress by iron, could compromise blood-brain barrier integrity and aggravate edema (Katsu et al., 2010; Urday et al., 2015).

Post-ICH hematoma expansion and rebleeding occur commonly in patients, suggesting a continuous enlargement of blood burden within the brain (Brott et al., 1997; Morgenstern et al., 2001). To counteract it, erythrophagocytosis by M φ occurs at both the edge and the center of hematoma, resulting in hematoma reduction (Cao et al., 2016). Timely clearance of hematoma can maintain homeostasis in cerebrovascular units and allows neurological recovery after ICH (Zhao et al., 2009). As such, shrinking hematoma via boosting the endogenous removal of RBCs, the source of hemolysis, has attracted increasing scientific interest in the past decade.

## Macrophage/Microglia: The Major Cell Performing Erythrophagocytosis

At onset, ICH rapidly recruits microglia, neutrophil, monocytes, and T lymphocytes, successively (Xue and Yong, 2020). For hematoma clearance, microglia and monocyte-derived macrophage are ‘professional’ phagocytes uptaking the damaged cells, including RBCs, in ICH (Xu et al., 2020; Li Q. et al., 2021). Though some ‘non-professional’ cells, such as endothelial cells, are also involved in erythrophagocytosis in cerebral microbleeds, their roles in ICH remain unknown (Chang R. et al., 2018). Microglia are the brain resident macrophages, mediating diverse functions critical to brain development and injury, such as synaptic pruning and phagocytosis (Paolicelli et al., 2011). For macrophage, their precursor cells – monocyte – are thought to enter the brain as a component of blood at ICH onset while later migrate into the brain via cell adhesion pathways (Engelhardt, 2008; Mracsko et al., 2014). These peripheral monocytes might undergo *in situ* differentiation into mature macrophages in the ischemic brain (Gliem et al., 2012; Miro-Mur et al., 2016). Interestingly, a recent study found that skull and vertebral bone marrow also supplied monocytes, which infiltrated the brain border (e.g., the dura matter) and developed into macrophages in inflamed brains (Cugurra et al., 2021). Given the limited evidence on ICH, a review on the dynamic infiltration of monocyte-derived macrophage in the ischemic brain might shed light on future studies (Han et al., 2020). Moreover, perihematomal M φ might follow the route of an available scaffold - white matter fibers – and migrate into the hematoma core, aiding the hematoma clearance (Chen et al., 2021). In addition to the white matter, molecules from the scar tissue are also considered able to support M φ activity, including phagocytosis, in brain injuries (Rolls et al., 2009).

**FIGURE 2:**
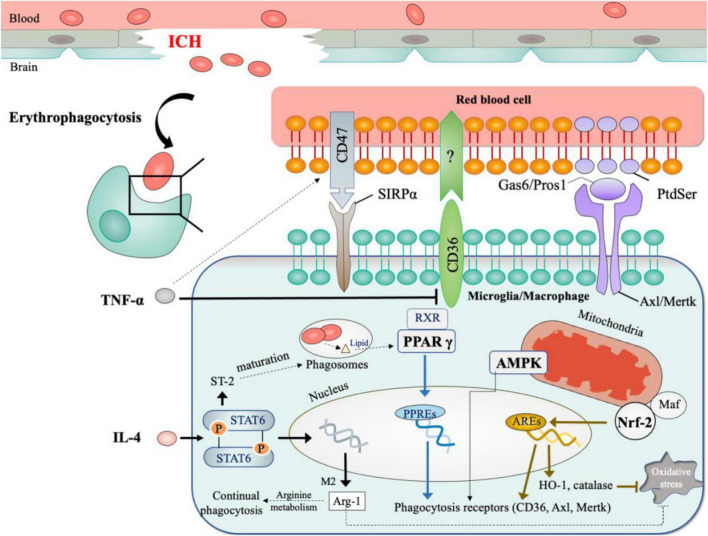
Mechanisms of erythrophagocytosis in ICH. **(1)** Signals for recognition include: CD47 on erythrocytes serves as the inhibitory signal for erythrophagocytosis; CD36, Axl, and Mertk on phagocytes act as scavenger receptors for clearing erythrocytes. **(2)** Microenvironment factors (inflammatory cytokines and oxidative stress) shape the phagocytosis by M φ. **(3)** The critical modulators for phagocytosis include PPAR γ, Nrf2, AMPK. *The dotted lines showed results from other diseases models.

Notwithstanding the diverse functions of macrophage and microglia observed in other types of strokes, most ICH studies could not distinguish macrophage from microglia due to the obstacles in differentiating between the two cell types *in vivo* (Zarruk et al., 2018). Fortunately, with the help of more specific cell markers (e.g., Tmem119 for microglia) and multi-channel flow cytometry (Bennett et al., 2016; Li et al., 2018), some researchers had begun to study the two cell types separately in ICH (Chang C. F. et al., 2018; Li Q. et al., 2021). Due to the inconsistent gating strategy applied by these two studies, it remains inconclusive as to how the role of macrophage differs from that of microglia. Therefore, in this review, we use M φ to denote the two cell types except when discussing studies that clearly distinguish between the two. Figure 2 summarizes the key findings on erythrophagocytosis after ICH.

## Membrane Receptors Initiate Erythrophagocytosis

Exogenous stimulus, such as inflammation, could impair erythrocytes integrity and predispose them to become apoptosis-like cells (Lang et al., 2014). The apoptotic erythrocytes are then recognized and phagocytosed by M φ, a process termed efferocytosis (Trahtemberg and Mevorach, 2017). Erythrophagocytosis in ICH is a type of efferocytosis. Blocking the M φ receptors for recognizing the apoptotic markers can impair the erythrophagocytosis and impede hematoma resolution. Efficient efferocytosis is important for tissue homeostasis by reducing exposure to toxic components of hemolysis and self-antigens which can substantially induce tissue damage and autoimmune response (Sisirak et al., 2016; Herzog et al., 2019). In ICH, efficient efferocytosis leads to controlled clearance of damaged erythrocyte before injuries are inflicted by uncontrolled hemolysis (Chang C. F. et al., 2018).

As a type of efferocytosis, erythrophagocytosis is a highly orchestrated process which can be separated into four consecutive steps (Hochreiter-Hufford and Ravichandran, 2013; Doran et al., 2020):

(1) sensing: dying cells release “find-me” signals to attract phagocytes;
(2) recognition: aged or abnormal erythrocytes (apoptotic erythrocytes) externalize the “eat-me” signals, such as phosphatidylserine (PtdSer) (de Back et al., 2014; Klei et al., 2017); phagocytes upregulate specific surface receptors (e.g., CD36 and TAM family) to recognize the “eat-me” signal on the dying cells; interestingly, apoptotic erythrocytes also express the “don’t eat me” signal, CD47, repelling M φ from efferocytosis (Ni et al., 2016); Table 1 summarizes the phagocytosis-related receptors by RBCs.
(3) ingestion: phagocytes initiate cytoskeleton rearrangement to facilitate internalization of the dying cells;
(4) digestion and response phase: phagocytes process the corpses and produce anti-inflammatory mediators to suppress inflammatory response.

**TABLE 1:** Receptors on the rede blood cells for erythrophagocytosis.

Function	Receptors	References
“Don’t eat-me” signal	CD47	ICH: Ni et al., 2016; Jing et al., 2019; Tao et al., 2020 Other diseases: Oldenborg, 2004
“Eat-me” signal	Phosphatidylserine (PtdSer)	ICH: Chang C. F. et al., 2018 Other diseases: Boas et al., 1998; Sun et al., 2021
	Band 3 clustering	Other diseases: Klei et al., 2017
	Calreticulin	Other diseases: Gardai et al., 2005; Nilsson et al., 2012

Of note, the current literature on erythrophagocytosis mainly focuses on the Step 3; the other steps remained largely unexplored in the context of ICH. Current studies have shown the therapeutic value of targeting the “don’s eat me signal” from erythrocytes (CD47) and the surface phagocytosis receptors from M φ (CD36 and TAM family).

### CD47

CD47 is an integrin-associated transmembrane protein ubiquitously expressed in many cell types including erythrocytes (Olsson et al., 2006). It regulates immune cell infiltration, phagocytosis, and the production of proinflammatory mediators and trophic factors by interacting with integrins and extracellular ligands (Brown and Frazier, 2001; Zhang et al., 2015). For phagocytosis, CD47 on erythrocytes acts as a “don’t eat me” signal to block phagocytosis by binding to signal regulatory protein α (SIRPα) on macrophage (de Back et al., 2014). Preclinical studies have demonstrated the role and therapeutic value of CD47 in ICH. The perihematomal level of CD47 increases within hours but decreases subsequently, accompanied by Mφ infiltration and erythrophagocytosis (Zhou X. et al., 2014; Cao et al., 2016). This explains the reverse correlation between CD47 and Mφ-mediated immune response. Intracranial injection of CD47 knock-out blood resulted in quicker hematoma resolution and milder brain edema (Ni et al., 2016). This effect was reduced by intracranial injection of clodronate liposomes, a specific phagocytes depletion drug. In all, CD47 may serve as an inhibitory signal in Mφ-mediated hematoma resolution. Inspiringly, CD47 blocking antibody significantly enhanced hematoma removal after ICH, rendering CD47 a promising and draggable target in ICH treatment (Jing et al., 2019; Tao et al., 2020).

### CD36

Upon activation by “eat-me” signals, macrophage upregulates several membrane scavenger receptors to direct the ingestion process, including CD36 (Silverstein and Febbraio, 2009). The upregulated precursor intracellular CD36 undergoes glycosylation in the endoplasmic reticulum, followed by transportation to the cell membrane (Alessio et al., 1996; Roszer, 2017). Membrane CD36 cooperates with the αvβ3 receptor to engage with the eat-me signal thrombospondin (TSP) on apoptotic cells, inducing the internalization and digestion of target cells (Savill et al., 1990, 1992).

In ICH, CD36-mediated apoptotic cells clearance is essential for hematoma resolution. Its transcription was upregulated in the erythrocytes-treated microglia culture as well as the perihematomal region after ICH (Zhao et al., 2007b). Both genetic deletion and antibody blocking of CD36 impeded the phagocytosis of erythrocytes by microglia (Zhao et al., 2007b; Fang et al., 2014). As M φ-mediated removal of erythrocytes is required for clot clearance, the roles and therapeutic values of CD36 in hematoma development has attracted considerable attention. For example, CD36 knock-out mice was found to have slower hematoma resolution and aggravated deficits when compared to wild-type mice after ICH (Fang et al., 2014). What’s more, the same study also found that patients with CD36 deficiency showed impaired hematoma resolution and poorer clinical outcome. Consistently, the upregulation of CD36 generates faster speed of erythrophagocytosis and hematoma resolution (Zhao et al., 2007b; Flores et al., 2016; Wang Y. et al., 2018). Enhancing the upstream regulatory mechanism of CD36, including peroxisome proliferator-activated receptor γ (PPAR γ) and the nuclear factor erythroid 2–related factor 2 (Nrf2), is a potential approach to promoting CD36-mediated hematoma clearance.

### Axl/Mertk

TAM – namely, Tyro3, Axl and Mertk – is a group of receptor tyrosine kinases functioning as macrophage scavenger receptor (Lemke and Burstyn-Cohen, 2010). Tyro3 is more highly expressed on neuron rather than on M φ in the brain, whereas Axl and Mertk are more abundant on M φ which makes them more relevant to erythrophagocytosis in ICH (Fourgeaud et al., 2016; Chang C. F. et al., 2018). Axl and Mertk participate in cell survival, migration, and phagocytosis by engaging with the ligands, growth arrest specific 6 (Gas6) and protein s (Pros1) (Shafit-Zagardo et al., 2018). Gas6/Pros1 acts by bridging Axl/Mertk with the “eat-me” signal (PtdSer) on apoptotic cells, initiating the phagocytosis process (Tondo et al., 2019).

In ICH, Axl, and Mertk might be required for erythrophagocytosis. In a murine model, the transcriptional level of Axl, Mertk and Gas6 ligand are increased within 24 h after ICH (Tong et al., 2017); deficiency of Axl/Mertk resulted in defective erythrophagocytosis by macrophage in ICH (Chang C. F. et al., 2018). However, results from double knockout Axl/Mertk were inconclusive as to whether both or either of them was responsible for these findings, it is likely that Mertk and Axl may have diversified functions, with Mertk playing important roles in homeostasis and Axl being more involved in inflammatory conditions (Zagorska et al., 2014). Further studies are necessary to define whether Axl or Mertk alone is indispensable for the removal of erythrocytes in ICH. Interestingly, Axl/Mertk also modulates M φ phenotypes and alleviates neuroinflammation in addition to its effects in phagocytosis. In ICH, Toll-like receptors (TLRs) polarizes M φ toward proinflammatory phenotype (M1), as opposed to anti-inflammatory (M2) phenotype (Lan et al., 2017). Axl/Mertk can activate the TLRs suppressors, SOC1 and SOC3, thereby inhibiting M1-like M φ activation and suppressing inflammatory response in a range of disease including in ICH (Rothlin et al., 2007; Tong et al., 2017; Chang C. F. et al., 2018; Wu et al., 2021).

From a therapeutic standpoint, exogenous ligands (e.g., recombinant Gas6) can be used to target Axl/Mertk-mediated beneficial effects. In inflammatory conditions, Axl/Mertk is prone to be cleaved from the cell membrane, generating the soluble but unfunctional Axl/Mertk (sAxl/sMertk). sAxl/sMertk competitively occupies the endogenous ligands (Gas6 and Pros1), resulting in the lack of ligands for regulating homeostasis in inflammatory conditions (Cai et al., 2016; Chang C. F. et al., 2018). Therefore, exogenous ligands, such as recombined Gas6 can compensate this insufficiency, serving as the druggable target in augmenting the effects of TAM (Di Stasi et al., 2020). For example, recombined Gas6 promoted inflammation resolution via Axl-dependent manner in experimental ICH (Tong et al., 2017). However, whether the exogenous ligands could facilitate the Axl/Mertk-mediated hematoma clearance warrants further investigation.

## Microenvironmental Factors Orchestrate the Erythrophagocytosis

Efficient phagocytosis requires phagocytes to digest multiple cells continuously, especially in acute inflammation where the apoptotic cells-to-phagocytes ratio is high (Doran et al., 2020). The significance of erythrophagocytosis in this context also depends on whether the M φ can remove such a tremendous amount of erythrocyte before irreversible hemolytic-induced brain injury commences (Bulters et al., 2018). Of relevance are microenvironmental factors, including neuroinflammation and oxidative stress, that may alter phagocytic function and therefore serve as viable therapeutic targets.

### Inflammatory Mediators

The correlation between M φ phenotypes and phagocytosis is complex. Erythrophagocytosis skewed M φ toward the M2 phenotype, which reciprocally facilitated the removal of dying cells (Bensinger et al., 2009; Roszer, 2017; Chang C. F. et al., 2018). It possibly explains for the protective roles of M2-M φ observed in both preclinical and clinical studies of stroke (Chernykh et al., 2016; Min et al., 2016; Bai et al., 2020). The cytokines involved in M1- and M2-M φ activation could modulate erythrophagocytosis in ICH.

Interleukin-4 (IL-4) is the canonical activator of signal transducer and an activator of transcription 6 (STAT6), which is essential to the activation of M2 phenotype (Lawrence and Natoli, 2011). In ICH, exogenous IL-4 activated STAT6 and enhanced erythrophagocytosis in animal after ICH (Xu et al., 2020). The article revealed two potential mechanisms. Firstly, IL-4/STAT6 was observed to upregulate CD36, the scavenger receptor initiating phagocytosis. This may result from the direct binding of STAT6 to the promotor regions of CD36 gene (Szanto et al., 2010). Secondly, the study proved IL-1 receptor like 1 (ST2) was required for IL-4/STAT6-mediated clearance of erythrocyte. As ST2 promoted phagosome maturation (Buckley et al., 2011), it is likely that IL-4/STAT6 regulated phagosome infusion and thus enhancing phagocytosis in ICH. It is important to note that IL-4/STAT6 transcriptionally upregulates the anti-inflammatory cytokines, which is likely to contribute to IL-4/STAT6 mediated pro-phagocytosis effect. For example, Arg1 is required for STAT6-mediated pro-phagocytosis by M φ in ischemic brain (Cai et al., 2019). The mechanism may link to the enzymatic role of Arg1 in arginine metabolism, which increased the M φ communication and resulted in continual phagocytosis (Yurdagul et al., 2020).

In the contrary, cytokines involved in M1-M φ activation are potential to inhibit the removal of apoptotic cells. Amongst, TNF-α is the potential cytokine for targeting. TNF-α stimulates the M1 phenotype and is also regarded as a marker of M1-M φ (Lan et al., 2017). In ICH, TNF-α downregulated CD36 in microglia and impaired its function in erythrophagocytosis (Fang et al., 2014). What’s more, TNF-α upregulated the “don’t eat-me” signal CD47 in vascular smooth muscle cells and renders less phagocytosis (Kojima et al., 2016). Thus, reverse the inhibition of TNF-α in erythrophagocytosis may be a potential approach to promote hematoma clearance. It is important to note that TNF-α inhibitors have been approved in the treatment of many diseases, such as ankylosing spondylitis and Crohn disease (Gerriets et al., 2021). However, whether these inhibitors can facilitate erythrophagocytosis and perform therapeutic effects remain unproved in ICH.

### Oxidative Stress

Oxidative stress is one of the major contributors toward pathological injury in ICH. The source of oxidative stress in ICH includes hemolytic products, mitochondria dysfunction, and M φ (Hu et al., 2016). Hemolytic products, particularly iron, catalyze a sequence of reactions known as the Haber-Weiss reaction, yielding highly reactive oxygen species (ROS) (Xiong et al., 2014). Mitochondria dysfunction allows abnormal leakage of electrons from electron transport chain, overwhelming antioxidant system and leading to accumulation of ROS. M φ also generates ROS by inhibiting oxidative metabolism (Zhou Y. et al., 2014) and processing large quantities by-products from the cell corpse (Splettstoesser and Schuff-Werner, 2002).

Oxidative stress have profound effects on M φ (Vernon and Tang, 2013). In M φ, ROS is essential for bactericidal effects, whereas it can kill the phagocytes when the levels becomes overwhelming (Morrow et al., 2007; van-Charvet et al., 2010). What’s more, the sudden onset of oxidative loading allows the transcription nuclear factor (NF)-κB to transfer from cytoplasm to nucleus, initiating the transcription of proinflammatory mediators including TNF-α (Brigelius-Flohe and Flohe, 2011; Sivandzade et al., 2019). As previously discussed, TNF-α is potential to block phagocytosis. Moreover, oxidants may alter cell structures or destroy the signals required for phagocytes (Anderson et al., 2002; Schrijvers et al., 2005). In all, oxidative stress may be a detrimental factor dampening normal function of M φ and compromising the removal of apoptotic cells.

Thus, the restriction of oxidative stress serves as a potential approach to improve M φ viability and facilitate erythrophagocytosis in ICH. To achieve this, strengthening the self-defense ability of M φ is a reasonable direction. To counteract the oxidative stress, M φ has developed the self-defense mechanisms with the mainstay represented by Nrf-2 (Virag et al., 2019). Treating microglia with the activator of Nrf-2 showed faster erythrophagocytosis speed (Zhao et al., 2015a). The roles of other self-defense mechanisms warrants more investigation. Besides, some substances that could sequestrates iron, the source of ROS, are also potent targets in facilitating erythrophagocytosis, such as lactoferrin. As a glycoprotein of transferrin family, lactoferrin was found to limit oxidative stress and promote microglia-mediated phagocytosis (Actor et al., 2009; Zhao et al., 2021). Lastly, Arg1, the M2 marker which is essential for arginine metabolism, could alleviate the oxidative stress by competing with inducible nitric oxide synthase (iNOS) for the arginine substrate (Corraliza et al., 1995; Morris and Jr, 2007). Given the role of arginine metabolism in phagocytosis (Yurdagul et al., 2020), this somehow reveals the internal links among inflammation, metabolism and oxidative stress in modulating phagocytosis.

## Upstream Regulators for Erythrophagocytosis

To initiate the erythrophagocytosis, the scavenger receptors in M φ drive the cell-to-cell interaction. These receptors are under controlled by by liver X receptor (LXR) and PPARs (α, β/δ and γ isotypes) (Roszer, 2017). Amongst, PPAR γ is the most studied regulator in ICH. Moreover, modulating the microenvironment factors shapes the function of M φ and enhances the phagocytosis efficacy. Nrf-2, as a powerful regulator of oxidative stress, has shown great therapeutic value in facilitating hematoma resolution and treating ICH. Last, energy metabolism is a critical component of efficient phagocytosis (Jiang et al., 2013). The regulatory role of AMPK, the energy sensor, in phagocytosis was also reviewed.

### PPAR γ

PPAR γ transcriptionally regulates genes that are critical to brain tissue repairment (Cai et al., 2018). Upon activation, PPAR γ heterodimerizes with retinoid X receptor (RXR) and subsequently engages with the conserved DNA sequences, namely peroxisome proliferator response elements (PPREs). PPREs is located in the promoter regions of cytoprotective genes, including the scavenger receptors and catalase, with the latter is essential to minimize oxidative injury (Cai et al., 2018). Thus, PPAR γ directly modulates phagocytosis and alleviates the oxidative stress, rendering it to be the most potent target in driving erythrophagocytosis and hematoma resolution in ICH.

In ICH, the activation of PPAR γ could upregulate the scavenger receptors and facilitate erythrophagocytosis. Generally, the scavenger receptors for clearing apoptotic cells are regulated by liver X receptor (LXR) and PPARs (α, β/δ and γ isotypes) with various combination patterns (Roszer, 2017): CD36 solely by PPAR γ, Axl by PPARs, and Mertk by PPARs and LXR. These patterns reflected the indispensable role of PPARs, especially PPAR γ, in regulating phagocytosis, which was supported by a number of ICH studies. Zhao et al. were the first to demonstrate that PPAR γ agonist could upregulate CD36 in microglia, thereby facilitating erythrophagocytosis *in vitro* (Zhao et al., 2007b). This pioneering work further proved that PPAR γ activation enhanced hematoma resolution and functional recovery in ICH. It paved the way for targeting M φ-mediated hematoma resolution in ICH (Zhao et al., 2009). Subsequently, activation of PPAR γ was observed to upregulate other scavenger receptors, Axl and Mertk, and expedite hematoma resolution in ICH (Zhuang et al., 2020). Moreover, the effects of PPAR γ activation in erythrophagocytosis were also verified in other types of hemorrhagic stroke (Wu et al., 2011; Flores et al., 2016). By activating PPAR γ, several pharmacological agents were found to confer protective effects in experimental ICH (Wang Y. et al., 2018; Fu et al., 2020; Zhao et al., 2020; Zhuang et al., 2020). Taken together, PPAR γ activation is one of the mainstays of phagocytosis modulator in facilitating hematoma clearance after ICH.

In addition, PPAR γ activation also alleviates inflammation and oxidative stress in ICH. For instance, PPAR γ agonists reduce proinflammatory TNF-α and IL-1β expression (Zhao et al., 2007b). Mechanistically, PPAR γ could prevent their nuclear receptor corepressor from being cleaved from the genes of TNF-α and IL-1β (Ghisletti et al., 2007; Jennewein et al., 2008). For oxidative stress, PPAR γ reciprocally interacted with Nrf-2 and synergistically inhibited NFκB mediated-oxidative stress (Zhao et al., 2015c; Luo et al., 2021). Thus, PPAR γ activation may also alleviate the inflammation and oxidative stress, producing a phagocytosis-friendly microenvironment for M φ.

To magnify PPAR γ-mediated protective effects, both the endogenous and exogenous stimulators are viable targets. PPAR γ is initially activated by the cell corpse-derived lipids in M φ when phagocytosis commences (Kourtzelis et al., 2020). Interestingly, the PPAR γ activity of M φ could also be modulated by other engulfed elements in addition to apoptotic cells. For instance, M φ could uptake the functional mitochondria and humanin released by astrocytes, which in turn upregulated the expression of PPAR γ and its mediated phagocytosis in ICH (Jung et al., 2020). In addition, activating PPAR γ-targeted genes and PPAR γ-mediated hematoma resolution could also be achieved by activating its transcription partner, i.e., RXR (Chang et al., 2020; Ting et al., 2020). Lastly, a clinical trial using pioglitazone, a known PPAR-gamma agonist approved by FDA, has been conducted in ICH patients, aiming to reduce hematoma burden and improve outcomes (NCT00827892). Other nuclear receptors may also play a role in clearing apoptotic cells, such as LXR, retinoic acid receptor (RAR), and glucocorticoid receptor (GR) (Roszer, 2017). Their roles in ICH warrants further investigation.

### Nrf-2

Nrf-2 is the principal transcriptional factor protecting cells from endogenous and exogenous stress (Kensler et al., 2007). Upon activation, Nrf2 heterodimerizes with the Maf family and binds to the antioxidant response elements (AREs) located in the regulatory regions of cytoprotective genes. These genes include antioxidant agents, such as catalase, HO-1, SOD, etc. (Zhao and Aronowski, 2013). Growing evidence supports the beneficial roles of Nrf2 in ICH. For instance, the absence of Nrf2 in rodents resulted in worse oxidative injury and more neurological deficits while the activation of Nrf2 reversed these effects in ICH (Wang et al., 2007; Zhao et al., 2007a; Iniaghe et al., 2015; Cheng et al., 2021).

Nrf-2 activation has also been proved to enhance the erythrophagocytosis by microglia and hematoma clearance in ICH (Zhao et al., 2015a). The effect of Nrf2 in promoting phagocytosis is partly CD36-dependent. The modulation of Nrf2 on CD36 may be PPAR γ-dependent or PPAR γ -independent, but this has not yet been confirmed (Ishii et al., 2004; Zhao et al., 2015c). Moreover, Nrf-2 regulates the genes responsible for detoxifying the blood products, including genes of ferritin, hemopexin and haptoglobin (Zhao and Aronowski, 2013; Deng et al., 2020; Liu et al., 2021) Nrf2 also regulates NF-κB pathway and alleviates inflammation in ICH, rendering targeting Nrf2 as a highly promising approach to augmenting hematoma resolution (Cheng et al., 2021). Indeed, agonists of Nrf2, such as sulforaphane, dimethyl fumarate and others, have been shown to accelerate hematoma reduction (Zhao et al., 2015a,b).

### AMP-Activated Protein Kinase

The reprograming of energy metabolic pathways is an emerging hallmark of anti-inflammatory (M2) M φ (Devanney et al., 2020). Particularly, these anti-inflammatory cells exhibit increased mitochondria oxidative metabolism with much lower level of glycolysis, pinpointing the demand for efficient energy production for inflammation resolution and tissue repair. AMP-activated protein kinase (AMPK) attracts particular interest in fulfilling this energy demand of M φ (Jiang et al., 2013). As an intracellular sensor of the “fuel status”, AMPK is activated by the drop of ATP-to-ADP ratio, which occurs in conditions like mitochondria inhibition, exercise, and starvation (O’Neill and Hardie, 2013; Herzig and Shaw, 2018). To counteract energy insufficiency, AMPK switches on catabolic activities to generates energy more efficiently while dampening anabolic process that consumes ATP. Given the intimate connection between cellular metabolism and immune response, the role of AMPK in macrophage functions has been reviewed (O’Neill and Hardie, 2013).

AMP-activated protein kinase activation has been reported to skew macrophage activation toward the M2 phenotype and enhance hematoma resolution in ICH (Vaibhav et al., 2018). Several *in vitro* studies also demonstrated that AMPK activation contributed to the M2 M φ polarization and phagocytosis (Wan et al., 2013; Jin et al., 2014; Kaiser et al., 2020; Seneviratne et al., 2020). Consistently, the absence of AMPK- α1, the sole isoform expressed in M φ, impaired the anti-inflammatory and prophagocytic effect of M φ in various brain disorders (Vaibhav et al., 2018; Lv et al., 2020).

The mechanisms underlying AMPK’s pro-phagocytosis effect are complex. On the one hand, AMPK cooperates with the transcriptional regulators (PPAR γ and Nrf-2), modulating their downstream scavenger receptors including CD36 and Mertk (Wan et al., 2013; Galvan et al., 2014; Wang Y. J. et al., 2018; Kaiser et al., 2020; Lv et al., 2020). On the other hand, AMPK promotes the organization of cytoskeleton and the acidification of endosomal-lysosomal system, facilitating the ingestion and digestion of apoptotic cells (Labuzek et al., 2010; Bae et al., 2011). In sum, AMPK activation favors inflammation resolution and might enhance apoptotic cells clearance in ICH.

Pharmacological agents, such as AdipoRon and CTRP3, also showed potential in driving AMPK-mediated augmentation on phagocytosis or inflammation resolution and functional recovery in preclinical ICH studies (Wang et al., 2016; Zheng et al., 2019). Several clinical trials on RIC-mediated AMPK activation and hematoma clearance are ongoing: NCT04757597, NCT04657133, NCT03930940 and NCT03481777. However, the direct evidence on the pro-phagocytotic effects of AMPK activation warrants verification in ICH.

## Future Directions

Given the limited evidence available on ICH in this context, studies in other types of stroke could inspire the future research direction in ICH. Ischemic stroke is the main type of stroke and therefore has drawn greater scientific interest than other types of strokes. Regarding the phagocytosis of apoptotic cells (including injured RBCs and neurons), the underlying mechanisms appear to be similar and comparable between the two conditions. For example, the scavenger receptor CD36, which was widely studied in ICH, has also been proved to mediate phagocytosis in ischemic stroke (Woo et al., 2016). The phagocytosis effects of other molecules, such as Arg-1 and PPAR γ, were also reported in ischemic strokes (Cai et al., 2019; Zhang et al., 2019).

Lastly, metabolic modulation, the phagocyted targets and the dynamic picture of pathophysiologic response after initial erythrophagocytosis are also important areas for future investigations.

### Better Understanding of the Metabolic Modulation in Erythrophagocytosis

One of the priorities of future studies was to explore the basic mechanisms of erythrocytes removal in the hemorrhagic brain. Although the basic biology of efferocytosis has been deeply studied, whether it could be applied to post-ICH erythrophagocytosis needs more investigation (Doran et al., 2020; Kourtzelis et al., 2020). Amongst, this review underlines the significance of defining the metabolic adaption of M φ which contributes to ongoing phagocytosis. Then, we could explore the potential targets for modulating this adaption to achieve efficient phagocytosis in ICH. Interestingly, the roles of metabolic modulation in phagocytosis might explain the neuroprotective effects of some approved agents. For instance, uncoupling protein 2 (UCP2), a mitochondria membrane protein, has been observed to improve neurological outcomes in stroke (Mattiasson et al., 2003; Nakase et al., 2007; Mehta and Li, 2009; Zhao et al., 2019). This protein maintained the membrane potential and supported continuous phagocytosis (Krauss et al., 2002, 2005; Park et al., 2011). However, whether UCP2 plays a role in facilitating phagocytosis in ICH is undefined.

### Better Understanding of the Phagocyted Targets

Erythrophagocytosis shares similar mechanisms in phagocytosing different apoptotic cells. Thus, enhancing the erythrophagocytosis signals can also eliminate other apoptotic cells nearby (Galloway et al., 2019). In ICH, the hematoma usually contains unvital neurons due to mechanical force and/or neurotoxicity of hematoma (Xi et al., 2006). Historically, eliminating the dying brain cells and synapses was regarded as a beneficial process since it would prevent self-antigen exposure and establish new homeostasis in the brain (review by Galloway et al. (2019)). However, the adverse effects of eliminating apoptotic neurons have recently been demonstrated in several brain disorders, including Alzheimer’s disease, multiple sclerosis, and strokes (Neher et al., 2013; Hong et al., 2016; Werneburg et al., 2020). The phagocytosis of “stressed-but-viable” neurons by M φ results in cell death, called “phagoptosis,” which could lead to delayed neuron loss (Brown and Neher, 2012; Neher et al., 2013). In ICH, a recent study demonstrated that the Mertk-dependent phagocytosis of synapse worsened the neurological outcome in animals after stroke (Shi et al., 2021). Table 2 outlines other adverse effects of phagocytosis which may be potentially contradictory to this review’s central tenet and suggest the need for a more specific phagocytosis system. Moreover, a systemic upregulation of erythrophagocytosis might disturb the physical removal of RBCs by splenic red pulp macrophage, since it involves the same phagocytosis-relevant receptors (Klei et al., 2017). Thus, future studies need to search for a pro-phagocytotic drug with localized specificity.

**TABLE 2:** Merits and Demerits of M φ-mediated phagocytosis.

Effects of phagocytosis	Targets	Details of effects
Merits	Red blood cells	Reduce clot toxicity and Support the functional recovery Zhao et al., 2009; Chang C. F. et al., 2018
	Neurons	Reduce inflammatory cytokines Lecca et al., 2018; favor neurogenesis Sierra et al., 2010.
	Synapse	Favor remyelination Natrajan et al., 2015.
Demerits	Red blood cells	Ferroptosis of phagocytes Youssef et al., 2018
	Neurons	Neuron death Brown and Neher, 2014; delayed neuron loss Neher et al., 2013; degeneration of dopaminergic neurons Barcia et al., 2013;
	Synapse	Synapse loss Hong et al., 2016; demyelination Han et al., 2012; worse functional outcome Shi et al., 2021.

## Conclusion: Erythrophagocytosis as a Spatiotemporally Developing Story

The foregoing review suggests that it is necessary to consider erythrophagocytosis in ICH as a spatiotemporally progressing event. There are mainly two concerns. Firstly, how could the M φ completely remove the damaged RBCs in a situation where the latter vastly outnumbers the former? Moreover, the population of M φ might be further reduced by the primary injury or the intracellular iron toxicity (van-Charvet et al., 2010; Youssef et al., 2018). To prevent the overwhelming of phagocytotic machinery, the number of M φ must be regulated to obtain sufficient phagocytotic capacity (Morioka et al., 2019). Therefore, therapies aimed at maximizing the number of functional phagocytes could improve RBC removal and should become a focus of future research. Inspiringly, this idea of introducing more functional M φ has recently been found to facilitate animal recovery from traumatic brain injury (Li Z. et al., 2021). Another layer of complexity lies in the fact that these immune cells tend to execute time-dependent effects in cerebrovascular injury, i.e., worsening the brain injury at acute phase but repairing the tissue at later stage (Mastorakos et al., 2021). One should therefore also take into consideration the optimal therapeutic time window when attempting to bring these cells into the brain. Secondly, what is the fate of M φ after engulfing RBCs in ICH? Although M φ could digest RBCs into the degradation products within themselves, could they process the blood components, especially iron, in a non-toxic way? Microglia contains the system for transporting and storing the iron in the normal brain, which is critical to the brain iron cycle and homeostasis (Winn et al., 2020). However, it remains unknown whether microglia could maintain its own homeostasis rather than undergoing iron-dependent cell death, namely “ferroptosis” in ICH (Youssef et al., 2018). Further studies are needed to achieve a better understanding of erythrophagocytosis and translate its therapeutic value to clinical practice.

## Author Contributions

JL and ZZ wrote the manuscript. GL supervised the drafting and revision of the manuscript. All authors contributed to the article and approved the submitted version.

## Conflict of Interest

The authors declare that the research was conducted in the absence of any commercial or financial relationships that could be construed as a potential conflict of interest.

## Publisher’s Note

All claims expressed in this article are solely those of the authors and do not necessarily represent those of their affiliated organizations, or those of the publisher, the editors and the reviewers. Any product that may be evaluated in this article, or claim that may be made by its manufacturer, is not guaranteed or endorsed by the publisher.
